# Cytoplasmic and Nuclear Anti-Apoptotic Roles of αB-Crystallin in Retinal Pigment Epithelial Cells

**DOI:** 10.1371/journal.pone.0045754

**Published:** 2012-09-26

**Authors:** Woo Jin Jeong, Jee Hyun Rho, Young Geol Yoon, Seung Hee Yoo, Na Young Jeong, Won Yeol Ryu, Hee Bae Ahn, Woo Chan Park, Sae Heun Rho, Hee Seong Yoon, Yung Hyun Choi, Young Hyun Yoo

**Affiliations:** 1 Department of Ophthalmology, Dong-A University College of Medicine, Seo-gu, Busan, Republic of Korea; 2 Department of Anatomy and Cell Biology, Dong-A University College of Medicine and Mitochondria Hub Regulation Center, Seo-gu, Busan, Republic of Korea; 3 Sungmo Eye Hospital, Inc., Haeundae-gu, Busan, Republic of Korea; 4 Department of Biochemistry and Research Institute of Oriental Medicine, Dongeui University College of Oriental Medicine, Busanjin-gu, Busan, Republic of Korea; University of Regensburg, Germany

## Abstract

In addition to its well-characterized role in the lens, αB-crystallin performs other functions. Methylglyoxal (MGO) can alter the function of the basement membrane of retinal pigment epithelial (RPE) cells. Thus, if MGO is not efficiently detoxified, it can induce adverse reactions in RPE cells. In this study, we examined the mechanisms underlying the anti-apoptotic activity of αB-crystallin in the human retinal pigment epithelial cell line ARPE-19 following MGO treatment using various assays, including nuclear staining, flow cytometry, DNA electrophoresis, pulse field gel electrophoresis, western blot analysis, confocal microscopy and co-immunoprecipitation assays. To directly assess the role of phosphorylation of αB-crystallin, we used site-directed mutagenesis to convert relevant serine residues to alanine residues. Using these techniques, we demonstrated that MGO induces apoptosis in ARPE-19 cells. Silencing αB-crystallin sensitized ARPE-19 cells to MGO-induced apoptosis, indicating that αB-crystallin protects ARPE-19 cells from MGO-induced apoptosis. Furthermore, we found that αB-crystallin interacts with the caspase subtypes, caspase-2L, -2S, -3, -4, -7, -8, -9 and -12 in untreated control ARPE-19 cells and that MGO treatment caused the dissociation of these caspase subtypes from αB-crystallin; transfection of S19A, S45A or S59A mutants caused the depletion of αB-crystallin from the nuclei of untreated control RPE cells leading to the release of caspase subtypes. Additionally, transfection of these mutants enhanced MGO-induced apoptosis in ARPE-19 cells, indicating that phosphorylation of nuclear αB-crystallin on serine residues 19, 45 and 59 plays a pivotal role in preventing apoptosis in ARPE-19 cells. Taken together, these results suggest that αB-crystallin prevents caspase activation by physically interacting with caspase subtypes in the cytoplasm and nucleus, thereby protecting RPE cells from MGO-induced apoptosis.

## Introduction

Crystallins found in non-lens tissues were predicted to be entirely different from those in the lens [1]–[3]. Previous studies on *in vivo* and *in vitro* expressed αB-crystallin suggest that it may function as an anti-apoptotic protein in human RPE cells [4], [5].

Methylglyoxal (MGO) is produced by various biochemical pathways and is present under normal physiological conditions in all biological systems [6], [7]. MGO contributes to the formation of advanced glycation end products (AGEs), reacts rapidly with RNA and denatured DNA, has both mutagenic and clastogenic activities [8]. Moreover, the accumulation of AGEs in RPE basement membrane is an acknowledged contributor to AMD [9]. Therefore, MGO can induce numerous adverse reactions if it is not efficiently detoxified [10], [11], and it is known to induce apoptosis in various cell types [12]–[15].

The fluorescent molecule bisretinoid is formed as a byproduct of vitamin A cycling in the retina, and as we age, it accumulates as lipofuscin in RPE cells [16]. Photochemical reactions initiated by these bisretinoid pigments contribute to the pathogenesis of several diseases that can threaten vision [17], [18]. Furthermore, photocleavage of A2E can produce MGO [18], and heat shock protein including alpha-crystallin which are susceptible to various post-translational modifications are particularly vulnerable to MGO-mediated modification [19], [20].

Therefore, the aim of this study was to examine whether MGO exposure induces apoptosis in RPE cells by interfering with the anti-apoptotic activity of αB-crystallin. We found that MGO induces apoptosis and that αB-crystallin exerts its anti-apoptotic functions by binding to caspase subtypes in the cytoplasm and nuclei of RPE cells.

**Figure 1:**
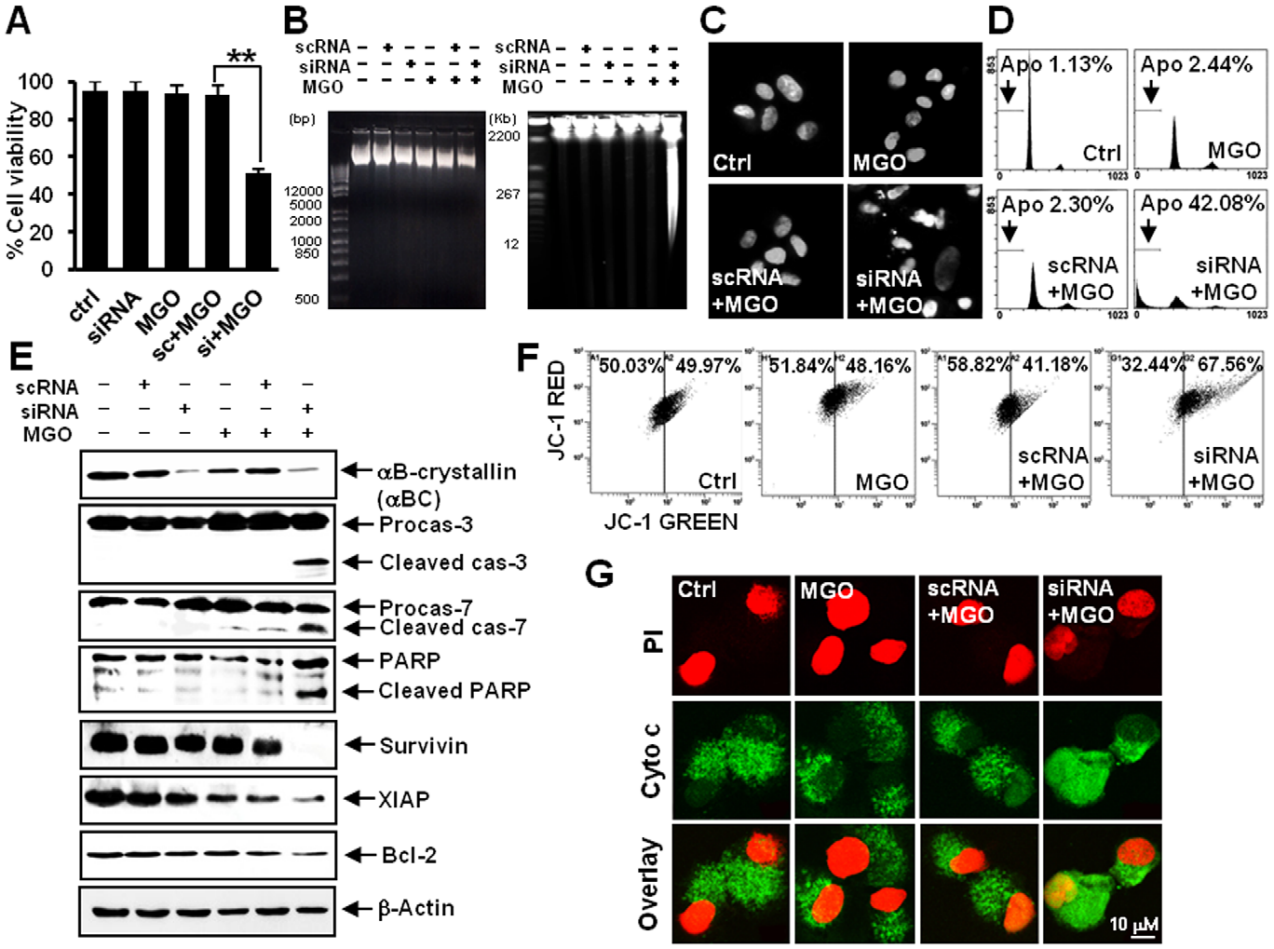
αB-crystallin protects ARPE-19 cells from MGO-induced apoptosis. ARPE-19 cells treated with 1 mM MGO for 4 h were harvested 48 h post-treatment (scRNA, scrambled siRNA; siRNA, RNAi against αB-crystallin; Ctrl, control). (A) Viability assay. Neither 1 mM MGO alone nor 1 mM MGO together with the scrambled siRNA control reduced the viability of ARPE-19 cells. However, silencing of αB-crystallin sensitized ARPE-19 cells and reduced their viability in the presence of 1 mM MGO. ** *P*<0.01. (B) DNA electrophoresis and PFGE. Whereas 1 mM MGO in conjunction with siRNA against αB-crystallin did not produce ladder-like DNA fragments on a conventional agarose gel (left), PFGE revealed the disintegration of nuclear DNA into giant fragments of 1–2 Mbp and high molecular-weight fragments of 100–1000 Kbp in length cells (right). (C) Nuclear morphology revealed by Hoechst staining. Silencing of αB-crystallin induced nuclear condensation of ARPE-19 cells in response to MGO. (D) Representative histograms indicating cell cycle progression and induction of apoptosis (Apo, the percentage of the population undergoing apoptosis). Silencing αB-crystallin induced the accumulation of subdiploid apoptotic ARPE-19 cells in response to MGO. (E) A western blot of apoptosis-related proteins. Silencing of αB-crystallin induced the degradation of procaspase-3 and -7 and PARP as well as the formation of their cleavage products following MGO treatment. In addition, downregulation of survivin, XIAP and Bcl-2 were observed (β-actin was used as a loading control). (F) Flow cytometry results indicating mitochondrial membrane potential (MMP). Silencing αB-crystallin induced a reduction in MMP in ARPE-19 cells in response to MGO. (G) Confocal microscopy images showing the subcellular localization of cytochrome c. Silencing αB-crystallin induced the release of cytochrome c from ARPE-19 mitochondria following MGO treatment. (PI, propidium iodide; Cyto C, cytochrome c).

## Materials and Methods

### Reagents

The following reagents were obtained commercially: polyclonal rabbit anti-human cytochrome c, caspase-2L and -2S, survivin, Bcl-2, RAIDD and PIDD as well as monoclonal mouse anti-human XIAP, hnRNP A1 and SF2/ASF antibodies from Santa Cruz Biotechnology (Santa Cruz, CA, USA); polyclonal rabbit anti-human αB-crystallin, phospho αB-crystallin-Ser19, -Ser45 and -Ser59 antibodies from Stressgen (Ann Arbor, MI, USA); monoclonal mouse anti-human poly (ADP-ribose) polymerase (PARP) antibody from Oncogene (Cambridge, MA, USA); polyclonal rabbit anti-human caspase-3, -4, -6, -7 and -12 and histone H3 antibodies as well as monoclonal mouse anti-human caspase-8 and -9 antibodies from Cell Signaling (Danvers, MA, USA); FITC-conjugated goat anti-rabbit and Texas Red-conjugated horse anti-mouse IgGs from Vector (Burlingame, CA, USA); HRP-conjugated donkey anti-rabbit and sheep anti-mouse IgGs from Amersham Pharmacia Biotech (Piscataway, NJ, USA); Dulbecco’s modified Eagle’s medium (DMEM) and fetal bovine serum (FBS) from Gibco BRL (Gaithersburg, MD, USA); polyclonal rabbit anti-human FLAG and monoclonal mouse anti-human SC35, α-tubulin and β-actin antibodies, Hoechst 33342, dimethylsulfoxide (DMSO), RNase A, proteinase K, aprotinin, leupeptin, propidium iodide (PI), phenylmethylsulfonyl fluoride (PMSF), protein-A agarose and methylglyoxal (MGO) from Sigma (St. Louis, MO, USA); caspase-2 inhibitor I (zVDVAD-fmk), caspase-3 inhibitor II (zDEVD-fmk), caspase-3 inhibitor IV (Ac-DMQD-CHO) and caspase inhibitor I (zVAD-fmk) from Calbiochem (San Diego, CA, USA); 5,5′,6,6′-tetrachloro-1,1′,3,3′-tetraethylbenzimidazol carbocyanine iodide (JC-1) from Molecular Probes (Eugene, OR, USA); and SuperSignal WestPico enhanced chemiluminescence western blotting detection reagent from Pierce (Rockford, IL, USA).

**Figure 2:**
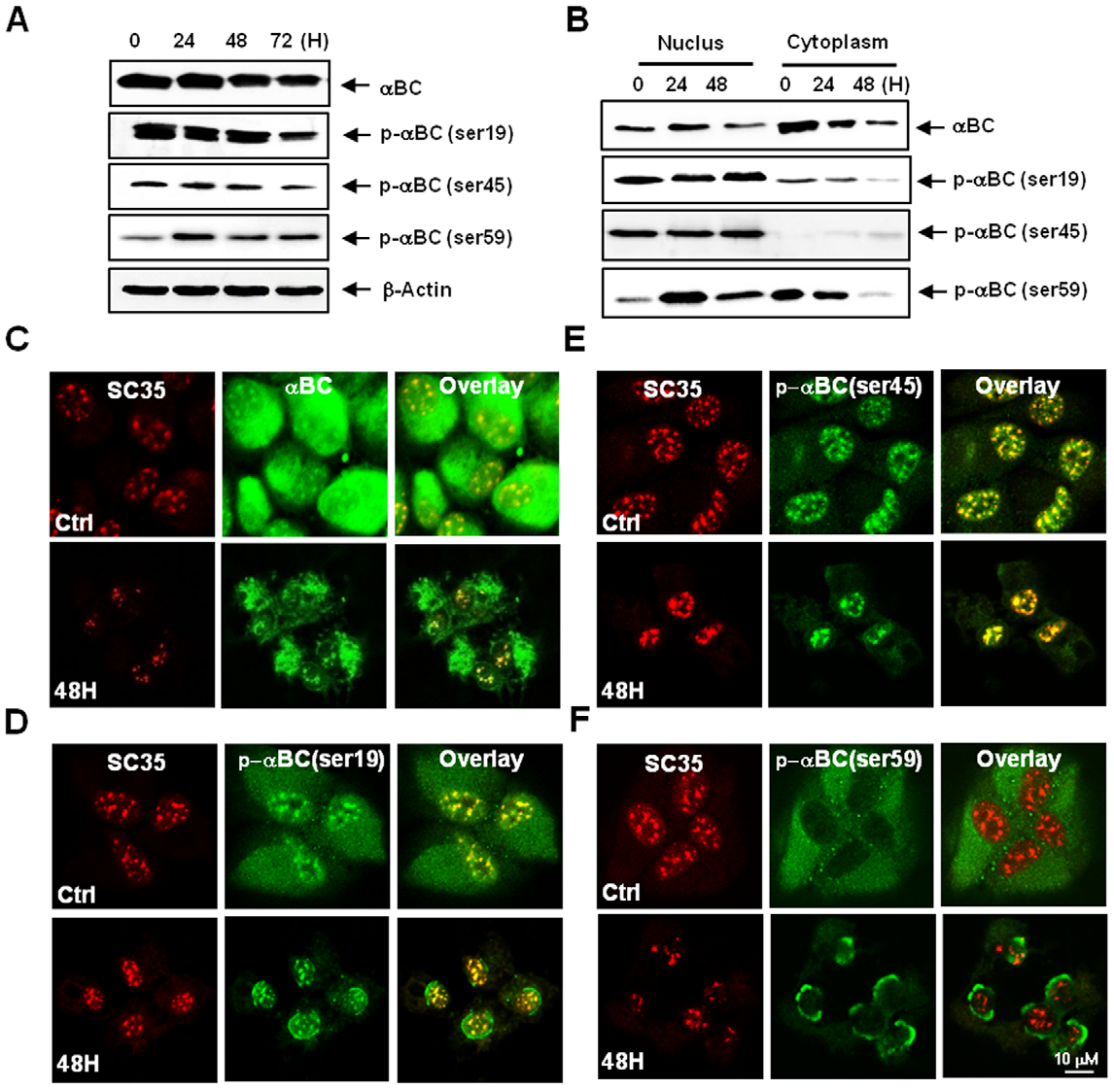
MGO modulates subcellular localization and phosphorylation of αB-crystallin at three different serine residues. ARPE-19 cells were treated with 2 mM MGO. (A) A western blot of αB-crystallin. Although MGO slightly decreased the expression of αB-crystallin, MGO downregulated the phosphorylation of αB-crystallin at Ser-19 and Ser-45. Conversely, MGO upregulated the phosphorylation of αB-crystallin at Ser-59 (β-actin was used as a loading control). (B) A western blot indicating the fractionation of cells treated with MGO. Cytoplasmic αB-crystallin was suppressed by MGO treatment, but nuclear αB-crystallin remained. P-αB-crystallin-Ser-19 was found in both the cytoplasm and nuclei of untreated control cells. MGO treatment caused the loss of cytoplasmic P-αB-crystallin-Ser-19, whereas its nuclear distribution remained unchanged. P-αB-crystallin-Ser-45 was found in the nuclei but not in the cytoplasm of untreated control cells, and its nuclear distribution remained following MGO treatment. P-αB-crystallin-Ser-59 was found primarily in the cytoplasm of untreated control cells, whereas MGO treatment increased the amount of nuclear P-αB-crystallin-Ser-59 while inducing its loss from the cytoplasm. (C–F) Confocal microscopy images showing the subcellular location of αB-crystallin and phosphorylation of αB-crystallin on Ser-19, Ser-45 or Ser-59. SC-35 and αB-crystallin were stained with Texas Red and FITC, respectively. (C) Confocal microscopy images showing αB-crystallin. αB-crystallin was localized to the cytoplasm and nuclei of untreated control ARPE-19 cells. Within nuclei, αB-crystallin was localized to SC35 speckles. MGO treatment substantially reduced cytoplasmic αB-crystallin, but the distribution of αB-crystallin in SC35 speckles was largely unaffected. (D) Confocal microscopy images of P-αB-crystallin-Ser-19. P-αB-crystallin-Ser-19 was found both in the cytoplasm and within nuclear SC35 speckles in untreated control cells. MGO treatment reduced cytoplasmic P-αB-crystallin-Ser-19, but its localization within SC35 speckles was unchanged. (E) Confocal microscopy images of P-αB-crystallin-Ser-45. P-αB-crystallin-Ser-45 was found in nuclear SC35 speckles in untreated control cells, and its distribution was unaffected by MGO treatment. (F) Confocal microscopy images of P-αB-crystallin-Ser-59. P-αB-crystallin-Ser-59 was found primarily in the cytoplasm of untreated control cells. MGO treatment reduced cytoplasmic P-αB-crystallin-Ser-59, causing its relocalization to the perinuclear region.

### Cell Culture and Treatment with MGO

ARPE-19 cells purchased from the American Type Culture Collection (Rockville, MD, USA) were cultured at 37°C under a 5% CO_2_ in air atmosphere, in a 1∶1 mixture of DMEM and Ham’s F12 medium with 10% FBS. Twenty-four hours after ARPE-19 cells were subcultured, the original medium was removed. Cells were washed with PBS and then incubated in the fresh medium as described above. MGO was added to the medium from a stock solution to a final concentration of 2 mM, and cells were incubated with the drug for 0, 24, 48, 72 or 96 h. Cells were harvested, stained with trypan blue and counted using a hemocytometer. We found that the concentration of PBS used in this study had no effect on ARPE-19 cell proliferation (data not shown).

### Caspase-7 siRNA

The caspase-7 siRNA (SMART pool; L-004407-00-0005) was purchased from Thermo Scientific (Waltham, MA, USA). As a negative control, we used a siRNA in which the same nucleotides were scrambled to form a combination not present in the genome.

**Figure 3:**
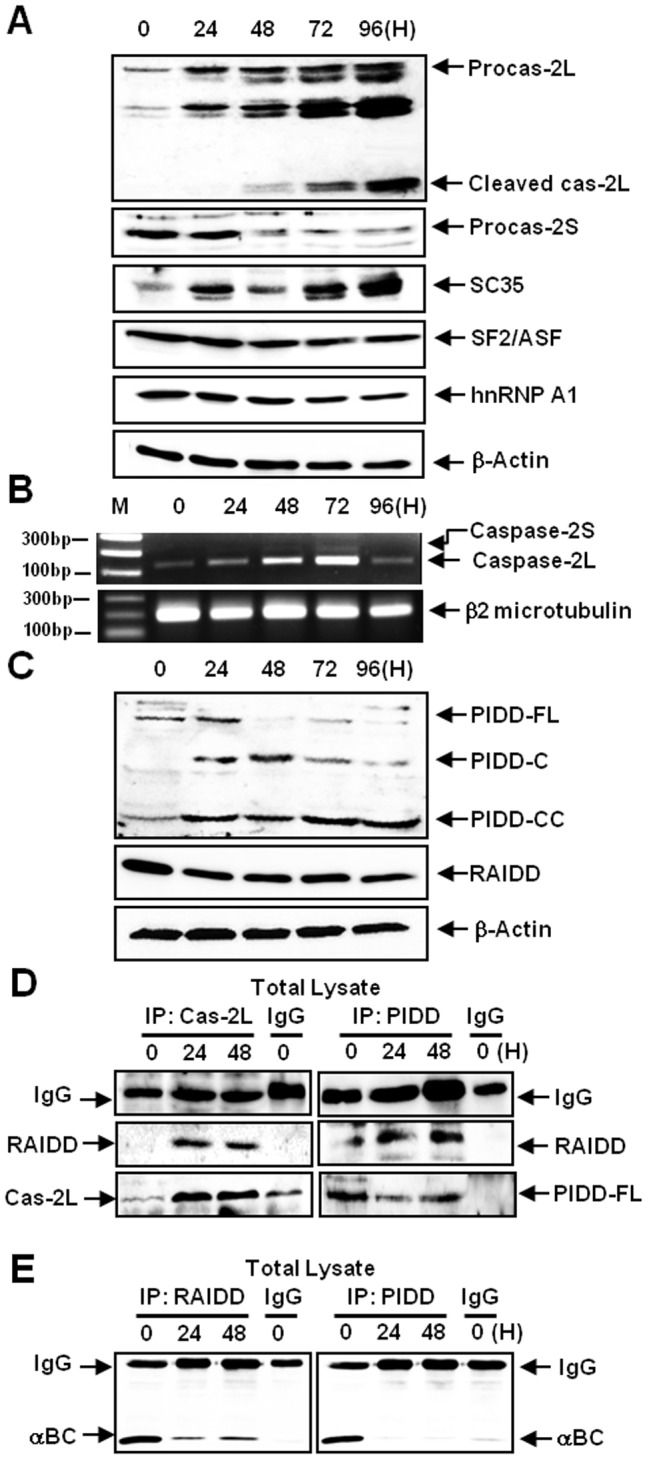
MGO-induced apoptosis is mediated by caspase-2 and PIDDosome formation. ARPE-19 cells were treated with 2 mM MGO. (A) A western blot showing the expression levels of several caspase-2 variants and splicing factors. MGO treatment triggered the production of the cleavage products of caspase-2L and -2S. Importantly, MGO upregulated the proform of caspase-2L, whereas MGO downregulated the proform of caspase-2S. MGO treatment substantially increased the expression SC-35 but slightly decreased the expression of SF2/ASF and hnRNP A1 (β-actin was used as a loading control). (B) An RT-PCR assay of caspase-2L and -2S expression. The expression levels of caspase-2L and -2S were increased by MGO treatment. The expression level of caspase-2L RNA transcripts was markedly increased in a time-dependent manner. (C) A western blot showing the expression of PIDDosome components. MGO treatment downregulated full-length PIDD but did not significantly alter levels of RAIDD expression. Importantly, MGO treatment upregulated PIDD-C and cause the production of PIDD-CC products (β-actin was used as a loading control). (D) Co-immunoprecipitation experiments demonstrating PIDDosome formation. Interaction between caspase-2L and RAIDD was observed following MGO treatment, whereas no interaction occurred between these proteins in untreated control cells. The interaction between PIDD and RAIDD was increased in cells treated with MGO. (E) A co-immunoprecipitation assay showing the interaction between αB-crystallin and RAIDD or PIDD. In the control ARPE-19 cells, αB-crystallin bound to RAIDD and PIDD, but these interactions were abolished by MGO treatment.

### αB-crystallin siRNA

A twenty one-nucleotide RNA with 3′-dTdT overhangs was synthesized by Dharmacon Research (Thermo Fisher Scientific, Lafayette, CO, USA) using the “ready-to-use” option. The AA-N19 mRNA targeted the αB-crystallin sequence (5′-AAUUGACCAGUUCUUCGGAGA-3′). As a negative control, we used a siRNA in which the same nucleotides were scrambled to form a combination not present in the genome.

**Figure 4:**
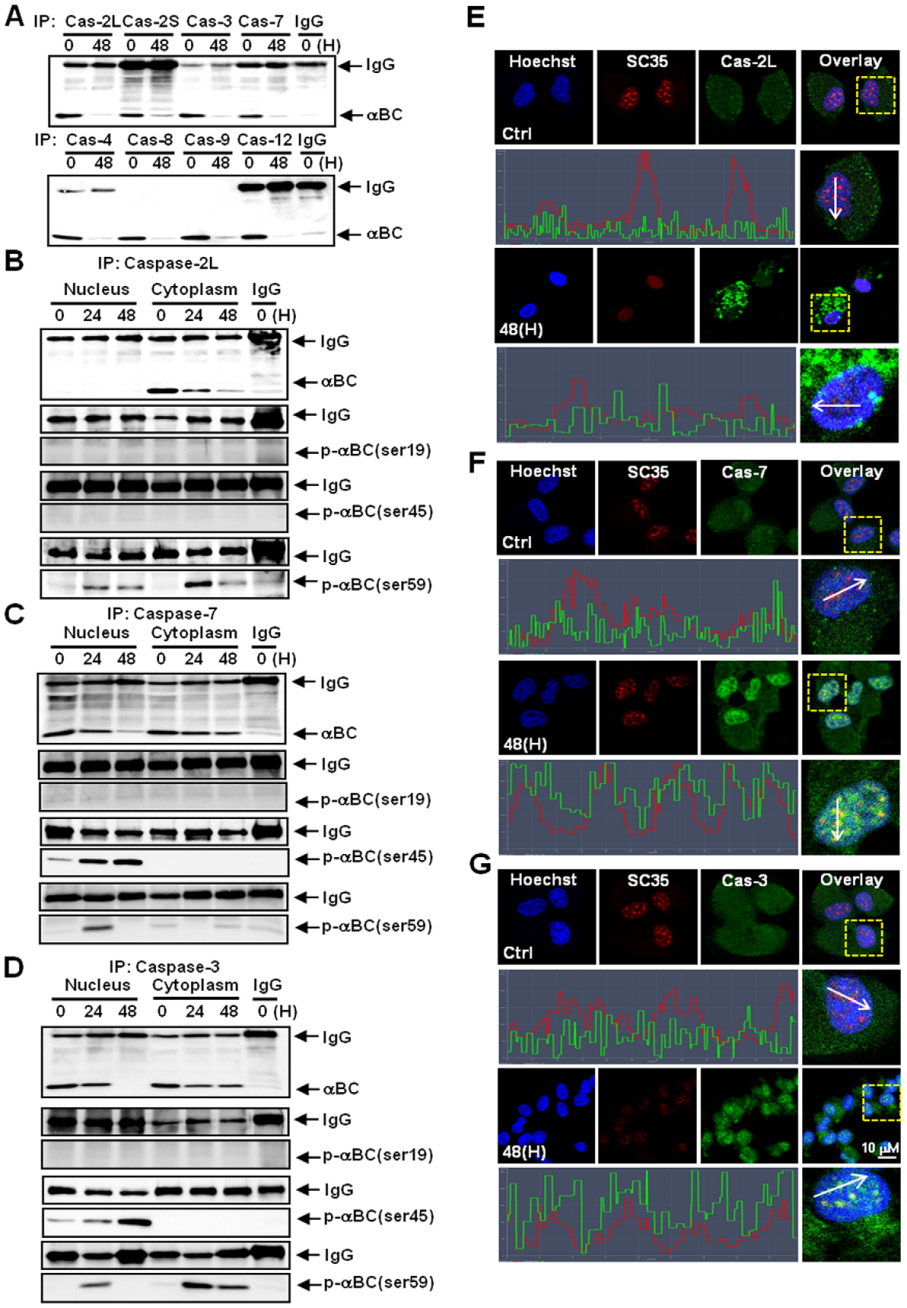
Interactions between αB-crystallin and various caspase subtypes. (A) A co-immunoprecipitation assay demonstrating the association between αB-crystallin and various caspase subtypes. All of the caspase subtypes tested interacted with αB-crystallin in the untreated control ARPE-19 cells. The interactions between αB-crystallin and each caspase subtype were reduced by MGO treatment. (B) A co-immunoprecipitation assay illustrating the interaction between αB-crystallin and caspase-2L; fractionated cell lysates (nuclear and cytoplasmic fractions) were used. Caspase-2L bound to αB-crystallin in the cytoplasmic fraction but not in the nuclear fraction of the untreated control ARPE-19 cells. The interaction between caspase-2L and un-phosphorylated αB-crystallin was abolished by treatment with MGO; this was evident at 48 h post-treatment. P-αB-crystallin-Ser19 and -Ser45 did not interact with caspase-2L in the untreated control cells or the MGO-treated cells. However, the interaction between P-αB-crystallin-Ser59 and caspase-2L was enhanced by MGO treatment in both fractions. (C) A co-immunoprecipitation assay indicating the interaction between αB-crystallin and caspase-7. Caspase-7 bound to unphosphorylated αB-crystallin in both the cytoplasm and nuclei of untreated control cells, but this interaction was decreased in both fractions when the cells were treated with MGO. P-αB-crystallin-Ser19 did not interact with caspase-7 in the untreated control cells or in MGO-treated cells in either fraction. P-αB-crystallin-Ser45 and -Ser59 interacted with caspase-7 in the nucleus, but not in the cytoplasm, and this interaction was found to be sustained or even increased following MGO treatment. (D) A co-immunoprecipitation assay indicating the interaction between αB-crystallin and caspase-3. Caspase-3 bound to unphosphorylated αB-crystallin both in the cytoplasm and the nucleus of untreated control cells, but this interaction was decreased in both fractions following MGO treatment. P-αB-crystallin-Ser19 did not interact with caspase-3 in untreated control cells or in MGO-treated cells. P-αB-crystallin-Ser45 interacted with caspase-3 in the nucleus, but not in the cytoplasm, and this interaction was found to be sustained or even increased following MGO treatment. P-αB-crystallin-Ser59 did not interact with caspase-3 in the untreated control cells in either fractions; however, an interaction was induced by MGO treatment, with a maximal effect observed at 24 h in both fractions. (E-G) Confocal microscopy images showing the association of caspase subtypes with SC35. The fluorescence intensity profiles of SC35 and each caspase subtype are depicted. (E) Caspase-2L is not located in the nuclei of ARPE-19 cells. (F) Caspase-7 is dispersed throughout the nuclei of the control cells. MGO treatment induced the localization of caspase-7 into SC35 speckles. (G) Caspase-3 is dispersed in the nuclei of control cells. MGO treatment induced the localization of caspase-3 into SC35 speckles.

### αB-crystallin siRNA Transfection Alone or in Combination with MGO Treatment

Transfections of siRNA were performed using siPORT Amine and Opti-MEM media. Cells grown to a confluence of 40–50% in six-well plates were transfected with siRNA at a final concentration of 100 nM per well. The transfection mixture was added to each well, and the cells were incubated for 4 h. Next, 2 ml of growth medium was added, and the cells were incubated for additional 20 h. Following removal of the siRNA transfection medium, each well was washed with PBS and the cells were treated with 1 mM MGO for 4 h. The treated cells were transferred to new media where they were incubated for an additional 48 h before harvesting.

### Cell Viability Assay

Cell viability was assessed by the Vi-Cell (Beckman Coulter, CA, USA) cell counter which performs an automated trypan blue exclusion assay.

### Nuclear Morphology Study for Apoptosis

Cell suspensions were cytospun onto clean lipid-free glass slides using a cytocentrifuge. Centrifuged samples were fixed for 10 min in 4% paraformaldehyde and stained with either 10 µg/ml PI or 4 µg/ml Hoechst 33342 for 30 min at 4°C. Cells were examined and photographed using an epifluorescence microscopy.

### Quantification of DNA Hypoploidy and Cell Cycle Phase Analysis by Flow Cytometry

Ice-cold 95% ethanol with 0.5% Tween 20 was added to the cell suspensions to a final concentration of 70% ethanol. Fixed cells were pelleted and washed in PBS with 1% BSA. Cells were re-suspended in 1 ml PBS containing 11 Kunitz U/ml RNase, incubated at 4°C for 30 min, washed once with PBS with 1% BSA, and re-suspended in PI solution (50 µg/ml). Following incubation at 4°C for 30 min in the dark, the cells were washed with PBS, their DNA content was measured on an Epics XL flow cytometer (Beckman Coulter, FL, USA). The data were analyzed using the Multicycle software program, which allowed us to perform simultaneous estimation of cell cycle parameters and apoptosis.

**Figure 5:**
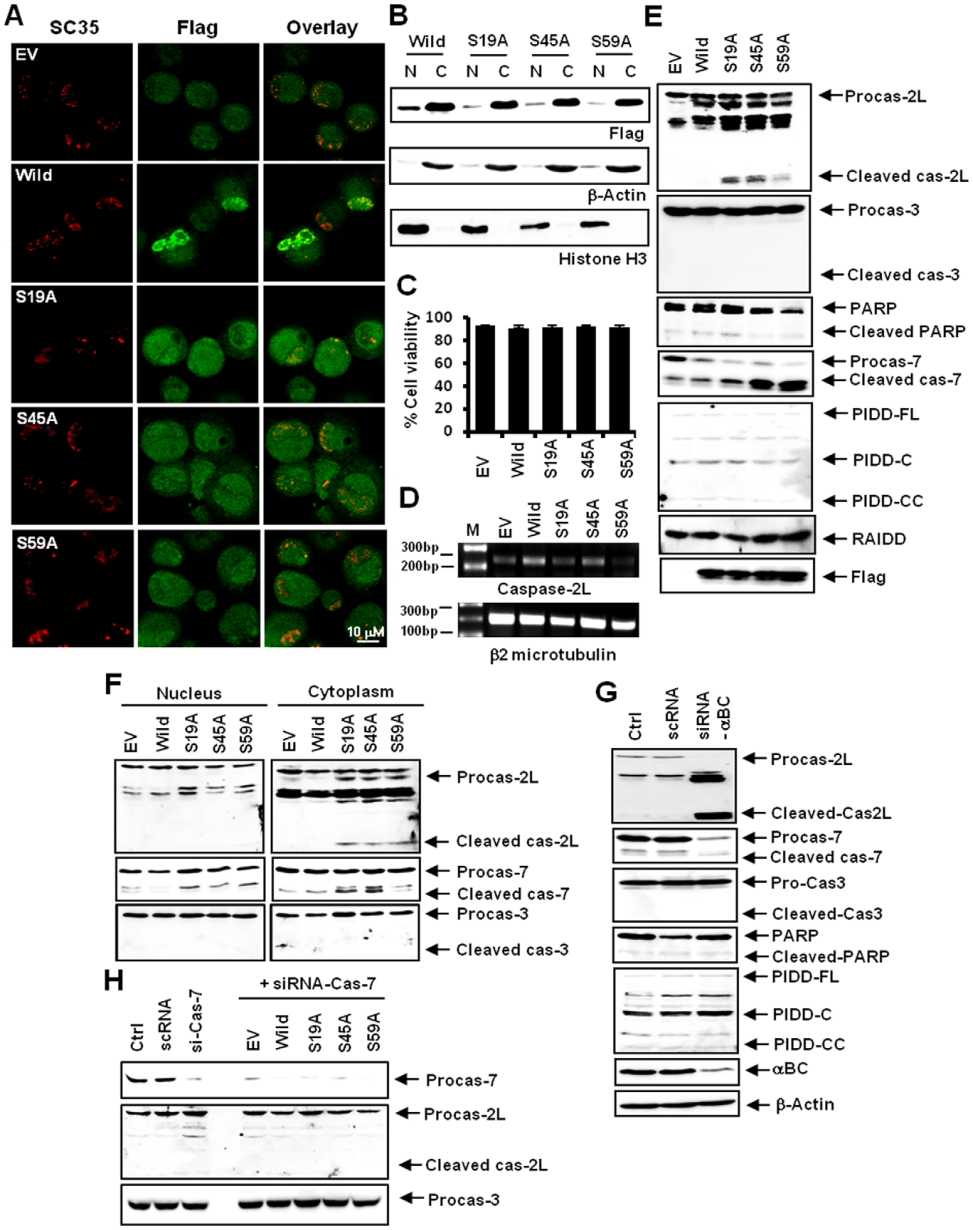
αB-crystallin inhibits the activation of caspase-2 and -7 in the nuclei of control ARPE-19 cells. ARPE-19 cells were transfected with constructs containing the wild-type αB-crystallin gene (*CRYAB*) or non-phosphorylatable *cryab* mutants (S19A, S45A or S59A). Following transfection, cells were treated with 2 mM MGO for 24 h, harvested and then assayed. (A) Confocal microscopy images showing that replacing residues Ser19, Ser45 or Ser59 with alanine prevented the nuclear localization of αB-crystallin in SC35 speckles. (B) A western blot indicating the localization of the nonphosphorylatable αB-crystallin mutants S19A, S45A and S59A. Because the αB-crystallin gene was fused to Flag sequences in the wild-type and mutant constructs, an anti-Flag antibody (Flag) was used to detect both wild-type and mutant proteins. Mutant αB-crystallin proteins were mostly retained in the cytoplasmic fraction (N, nuclear fraction; C, cytoplasmic fraction. β-actin and histone H3 were used as loading controls). (C) A viability assay. The introduction of αB-crystallin mutants did not affect the viability of cells. (D) An RT-PCR assay indicating that transfection of these mutants induced caspase-2L cleavage without concomitant caspase-2L upregulation. (E) A western blot. Transfection of the αB-crystallin mutants induced the formation of cleavage products from caspase-2L and -7, but not from caspase-3 or PARP. Transfection of the αB-crystallin mutants did not affect PIDD-CC and RAIDD formation (Flag, an anti-Flag antibody was used to detect the wild type and mutant αB-crystallin transfected into the cells). (F) A western blot showing the cleavage products of various caspase subtypes. Wild-type and mutant αB-crystallin constructs were transfected into cells and both nuclear and cytoplasmic fractions were isolated from the transfected cells. Cleaved caspase-7 was found in the both the cytoplasmic and nuclear compartments, whereas the cleavage products of caspase-2L appeared only in the cytoplasm. No cleavage product of caspase-3 was observed in either the cytoplasm or the nuclei. (G) A western blot indicating the effects of silencing αB-crystallin. siRNA against αB-crystallin efficiently reduced αB-crystallin expression, caused the production of a caspase-2L cleavage product, and induced the degradation of procaspase-7. However, no changes were observed in the expression or production of cleavage products for caspase-3, PARP or PIDD-CC (β-actin was used as a loading control). (H) A western blot indicating the effects of silencing caspase-7. Caspase-7 siRNA prevented the activation of caspase-2L in S19A, S45A or S59A mutant-expressing ARPE19 cells. Caspase-7 siRNA used in this experiment efficiently reduced the expression of caspase-7.

### DNA Electrophoresis

DNA from 10^6^ cells was loaded into each lane of a 2% agarose gels in Tris-acetic acid/EDTA buffer containing 0.5 µg/ml ethidium bromide (EtBr); gels were electrophoresed at 50 mA for 1.5 h. The DNA fragments were separated by 1.8% agarose gel electrophoresis and visualized under UV light.

### Pulsed Field Gel Electrophoresis (PFGE)

PFGE was carried out in 0.5**×** TBE maintained at 14°C by circulation of cool water over 16 h using the CHEF Mapper XA System from Bio-Rad (Hercules, CA, USA). DNA in the gels was stained with EtBr and detected using an LAS-3000 Plus Imager (Fuji Photo Film Company, Kanagawa, Japan). Chromosomal DNA from *Saccharomyces cerevisiae* and a mixture of λ DNA, its concatemers, and *Hind*III-digested λ DNA were used as DNA size markers.

**Figure 6:**
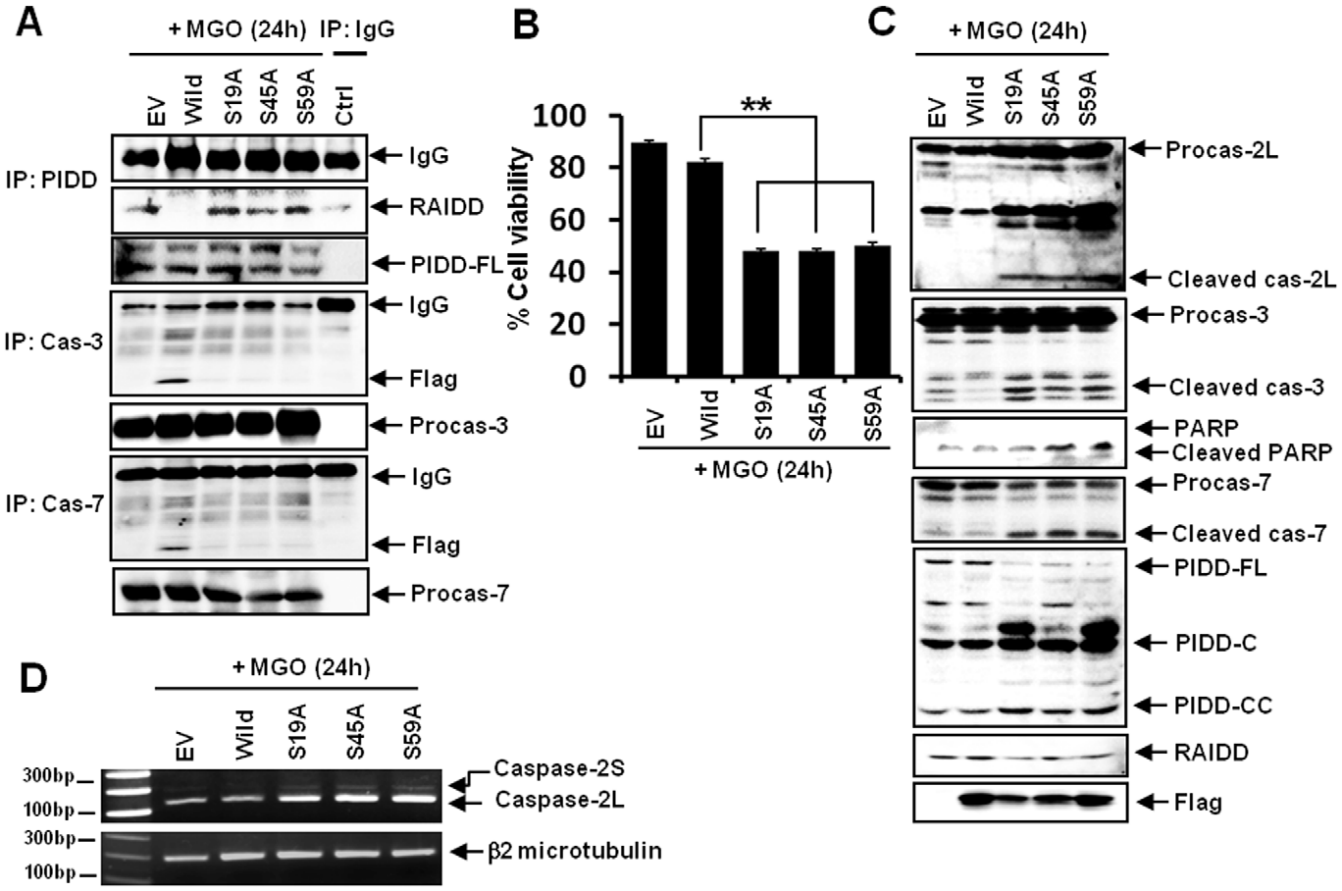
Phosphorylation of αB-crystallin at serines 19, 45 and 59 plays a pivotal role in preventing MGO-induced ARPE19 apoptosis. ARPE-19 cells were transfected with constructs containing wild-type or nonphosphorylatable αB-crystallin mutants (S19A, S45A or S59A). (A) A co-immunoprecipitation assay indicating the interactions between αB-crystallin and caspase-3 or -7 as well as between PIDD and RAIDD in mutant cells. While the interactions between αB-crystallin and caspase-3 or -7 were decreased, the interaction between RAIDD and PIDD was increased in mutant-expressing cells (Flag, Flag antibody used to detect the wild type and mutant αB-crystallin). (B) The viability of mutant cells treated with 2 mM MGO for 24 h. All mutant-expressing cells showed a significant reduction in viability at 24 h post-MGO treatment. ** *P*<0.01. (C) A western blot indicating the effects of mutant αB-crystallin expression in response to MGO treatment. MGO upregulated the expression of caspase-2L in mutant-expressing cells. The cleavage products of caspase-2L, -7 and -3, PARP, and PIDD-CC were also increased in the mutant cells (Flag, Flag antibody used to detect the transfected wild-type and mutant αB-crystallin). (D) RT-PCR assays showing the expression of caspase-2L in mutant-expressing cells. Caspase-2L mRNA transcripts were increased in the mutant cells treated with MGO.

### Western Blot Analysis

Equal amounts of proteins were separated using 7.5–15% sodium dodecylsulfate-polyacrylamide gel electrophoresis (SDS-PAGE). The protein from gels was transferred to nitrocellulose membranes (Amersham Pharmacia Biotech, Piscataway, NJ, USA) and incubated with each antibody. Immunostaining with antibodies was performed using SuperSignal WestPico enhanced chemiluminescence substrate and detected using an LAS-3000 Plus imager.

### Immunofluorescence Staining, Confocal Microscopy and Quantification

Cells were cultured on a coverslips and incubated with primary antibodies for 1 h at 37°C. Cells were then washed 3 times for 5 min each with PBS, incubated with a FITC-conjugated secondary antibody for 1 h at room temperature and counterstained with propidium iodide. For double-immunofluorescent stainings, cells were incubated with two different primary antibodies for 1 h at 37°C and washed three times for 5 min each with PBS. Secondary antibodies conjugated with either FITC or Texas Red were used. Fluorescence images were observed and analyzed using a Zeiss LSM 510 laser-scanning confocal microscope (Goettingen, Germany).

### Assay of Mitochondrial Membrane Potential (MMP)

To measure MMP, cells were stained with JC-1, and flow cytometry was performed using an Epics XL flow cytometer (Beckman Coulter, FL, USA). Data were acquired and analyzed using the EXPO32 ADC XL 4 color software program.

### Subcellular Fractionation

ARPE-19 cell (10^7^ cells/well) were washed in Tris-based Mg^2+^/Ca^2+^-deficient buffer (135 mM NaCl, 5 mM KCl, and 25 mM Tris pH 7.6) and allowed to swell for 10 min in ice-cold hypotonic CaRSB buffer (10 mM NaCl, 1.5 mM CaCl_2_, 10 mM Tris, pH 7.5, and 1× protease inhibitor cocktail). Cells were dounced with 60 strokes, and mitochondria stabilization buffer (210 mM mannitol, 70 mM sucrose, 5 mM EDTA, and 5 mM Tris pH 7.6) was added to stabilize the mitochondria. After collecting the nuclei by centrifugation twice at 3,000 rpm for 15 min, the supernatant was spun at 14,000 rpm for 20 min at 4°C; the pellet and supernatant included the mitochondrial and cytoplasmic fractions, respectively. Swollen cells (5×10^7^) in ice-cold hypotonic CaRSB buffer were dounce-homogenized, and the cell homogenates were spun down twice at 3,000 rpm for 15 min.

### Co-immunoprecipitation (Co-IP)

Cell extracts incubated with antibodies were precipitated with protein A-agarose beads. Aliquots of the immunoprecipitated proteins were separated by SDS-PAGE, and western blot analyses were performed as described above. The results of each co-IP experiment were confirmed via reciprocal IP (data not shown).

### Plasmid Constructs and Site-directed Mutagenesis

Full-length human αB-crystallin cDNA (*CRYAB*) (accession number: NM_001885) was purchased from 21 Century Human Gene Bank (21 Century Frontier Human Gene Bank, Taejeon, Korea) and subcloned into pcDNA6-3xFlag. The vector pcDNA6-3xFlag was constructed by inserting the 3xFlag tag sequence between the *Nhe*I and *Xho*I sites of pcDNA6 (Invitrogen, Carlsbad, CA, USA). The following primers were used: CRYABFwd (5′– AAGAATTCATGGACATCGCCATCCACCACCCC-3′) and CRYABRev (5′-TTTCTAGACTATTTCTTGGGGGCTGCGGTGACAGC -3′); the *Eco*RI and *Xba*I sites in CRYABFwd and CRYABRev, respectively, have been underlined. The PCR-amplified αB-crystallin gene was cloned between the *Eco*RI and *Xba*I sites, which generated an in-frame fusion with the 3xFlag tag. Site-directed mutagenesis was carried out using the Quick Change Site-Directed mutagenesis kit (Stratagene, La Jolla, CA, USA) with the following primers: S19AFwd (5′-TTCCTTTCCACGCCCCCAGCCGCC-3′), S19ARev (5′-AGGCGGCTGGGGCGTGGAAAGG-3′), S45AFwd (5′-TACTTCCCTGGCCCCCTTCTACCTTCGGCCACC-3′), S45ARev (5′-GTGGCCGAAGGTAGAAGGGGGCCAGGGAAGTAG-3′), S59AFwd (5′-CTGCGGGCACCCGCCTGGTTTGACACTG-3′) and S59ARev (5′-ATGGTCAAACCAGGCGGGTGCCCGCAGG-3′). All constructs were verified by nucleotide sequencing.

### Cell Transfection and Visualization

ARPE-19 cells grown in six-well plates (10^5^ cells/well) were transiently transfected with the green fluorescence protein (GFP) fusion αB-crystallin construct using Lipofectamine 2000 (Invitrogen) according to the manufacturer’s instructions. Briefly, 2 µg of plasmid DNA was mixed with 6 µl Lipofectamine 2000 and incubated with Opti-MEM. The plasmid DNA-Lipofectamine 2000 complex was added to the cells, which were then incubated for 4 h at 37°C. Following incubation, the medium was replaced with 2 ml growth medium, and the cells were incubated for an additional 20 h. Twenty-four hours after transfection, the plasmid-DNA transfection medium was removed, and each well was washed with PBS solution. Cells were treated with 2 mM MGO for 24 h. Intracellular localization of Flag-αB-crystallin was analyzed by the EGFP fluorescent detection method using a Zeiss LMS 510 laser-scanning confocal microscope (Göettingen, Germany).

### Reverse Transcriptase PCR (RT-PCR)

Total RNA was extracted from the cultured cells using Trizol reagent (Invitrogen) according to the manufacturer’s instructions, and total RNA concentrations were measured by spectrophotometry. Reverse transcriptase PCR was performed using the SuperScript III First Strand cDNA synthesis kit (Invitrogen). The resulting cDNAs were then used as templates for PCR amplification. PCR analysis of caspase-2 and β2 microtubulin was performed using gene specific primers (caspase-2∶ 5′-GTTACCTGCACACCGAGTCACG-3′ and 5′- GCGTGGTTCTTTCCATCTTGTTGGTCA-3′; β2 microtubulin: 5′-CTCACGTCATCCAGCAGAGA-3′ and 5′-TCTTTTTCAGTGGGGGTGAA-3′). The following PCR cycle parameters were used for the RT-PCR: 95°C for 3 min, followed by 32 cycles at 94°C for 1 min, 60°C for 1 min, and 72°C for 1 min, followed by a final 72°C step for 10 min; reactions were carried out using a GeneAmp PCR System 9700 Thermal Cycler (Applied Biosystems, Carlsbad, CA, USA). Amplified products were separated on a 1.2% agarose gel and purified using the QIA Quick Gel Extraction Kit (Qiagen, Valencia, CA, USA). Gel-purified PCR products were then sequenced.

### Statistical Analysis

Four independent experiments were carried out *in vitro*, and the results are expressed as the mean ± S.D. from four experiments, each performed in triplicate. The results of the experimental and control groups were tested for statistical significance using the Kruskal-Wallis nonparametric test.

## Results

### Methylglyoxal Induces Apoptosis in ARPE-19 Cells

We found that MGO efficiently reduced the viability of ARPE-19 cells at 1.5–3.5 mM concentrations in a dose-dependent manner (Fig. S1A). Because the viability of ARPE-19 cells treated with 2 mM MGO for 48 h was approximately 50% of control values, this concentration was used in all further studies. At 2 mM, MGO also reduced the viability of ARPE-19 cells in a time-dependent manner (Fig. S1B). Finally, following treatment with MGO, ARPE-19 cells exhibited apoptotic phenotypes (Fig. S2A–G).

### αB-crystallin Protects ARPE-19 Cells from MGO-induced Apoptosis

The effects of αB-crystallin knockdown were tested in ARPE-19 cells treated with a subtoxic dose of MGO. Neither 1 mM MGO alone nor 1 mM MGO with the scrambled control siRNA was found to reduce the viability of ARPE-19 cells. However, siRNA-mediated silencing of αB-crystallin sensitized ARPE-19 cells to 1 mM MGO, leading to a reduction in viability (Fig. 1A). Following co-treatment, ARPE-19 cells exhibited apoptotic phenotypes (Fig. 1B–G).

### MGO Modulates the Phosphorylation and Subcellular Localization of αB-crystallin

MGO treatment slightly decreased the expression of αB-crystallin (Fig. 2A). Cytoplasmic αB-crystallin was lost in response to MGO treatment, while nuclear localization of αB-crystallin remained (Fig. 2B); αB-crystallin was distributed in both the cytoplasm and nuclei of control ARPE-19 cells. In the nucleus, αB-crystallin was mainly localized to SC35 speckles (Fig. 2C, 0 h). MGO treatment induced the loss of a significant fraction of cytoplasmic αB-crystallin; in contrast, the nuclear distribution of αB-crystallin was not perturbed by MGO treatment (Fig. 2C, 48 h). In control cells, both P-αB-crystallin-Ser19 and P-αB-crystallin-Ser45 were abundant, whereas P-αB-crystallin-Ser59 was detected at only low levels (Fig. 2A, 0 h). MGO treatment downregulated both P-αB-crystallin-Ser19 and P-αB-crystallin-Ser45 while upregulating P-αB-crystallin-Ser59 (Fig. 2A). Post-fractionation western blotting and confocal microscopy demonstrated that P-αB-crystallin-Ser19 was localized to the cytoplasm and nuclei in control cells and that MGO treatment induced the evacuation of cytoplasmic P-αB-crystallin-Ser19 without perturbing its nuclear distribution (Fig. 2B, D). P-αB-crystallin-Ser45 was found in the nucleus but not in the cytoplasm of control cells, and its nuclear distribution was not altered by MGO treatment (Fig. 2B, E). P-αB-crystallin-Ser59 was primarily located in the cytoplasm of control cells, but was found to be reduced in the cytoplasm and substantially increased in the nucleus following MGO treatment (Fig. 2B). P-αB-crystallin-Ser59 localized mainly around the nuclear periphery following MGO treatment (Fig. 2F).

### MGO-induced Apoptosis is Mediated by Caspase-2 and PIDDosome Formation

Because αB-crystallin localizes to SC35 speckles that function as sites for storage and/or recycling of splicing factors, we next examined whether caspase-2 (which consists of two splice variants) is involved in MGO-induced apoptosis. MGO upregulated the proform of caspase-2L but downregulated the proform of caspase-2S (Fig. 3A). The expression of caspase-2L RNA transcripts was markedly increased, while the expression of caspase-2S was only slightly increased by MGO treatment (Fig. 3B). MGO affects the splicing machinery and alters the equilibrium of caspase-2 splicing variants (Fig. 3A). Furthermore, MGO treatment induced upregulation of the 51 kDa C-terminal fragment containing the death domain (PIDD-C) as well as the production of the 37 kDa C-terminal fragment (PIDD-CC); however, MGO treatment did not significantly alter the expression of RAIDD (RIP-associated Ichl-homologous protein with a death domain) (Fig. 3C). Co-immunoprecipitation experiments revealed that caspase-2L did not interact with RAIDD in the control cells. Importantly, MGO treatment induced an interaction between caspase-2L and RAIDD (Fig. 3D). Caspase-2L cleavage products were rare and difficult to observe at 24 h post-MGO treatment; however, the interaction between caspase-2L and RAIDD was evident at that time. These data indicate that the interaction between caspase-2L and RAIDD precedes the formation of caspase-2L cleavage products, suggesting that this interaction plays a role in caspase-2 activation. PIDD (p53-induced death domain-containing protein) and RAIDD also bound to one another when MGO was applied (Fig. 3D). We found that αB-crystallin interacted with RAIDD and PIDD in control cells (Fig. 3E, 0 h) and that MGO treatment abolished these interactions. Both RAIDD and PIDD were completely dissociated from αB-crystallin at 24 h post-MGO treatment, indicating that the release of RAIDD and PIDD from αB-crystallin precedes the upregulation of PIDD-C and the production of both the PIDD-CC and caspase-2L cleavage products (Fig. 3E).

### Dissociation of Caspases from αB-crystallin Correlates with Induction of Apoptosis

We next sought to determine which caspase subtype(s) interact with αB-crystallin in ARPE-19 cells. αB-crystallin interacted with all of the caspase subtypes tested in control cells, and MGO treatment decreased these interactions (Fig. 4A). Subsequent assays were conducted with specific focus on caspase-2L and two execution caspase subtypes: caspase-3 and -7 (Fig. 4B–D). Co-immunoprecipitation assays using fractionated cell lysates showed that αB-crystallin interacted with caspase-2L in the cytoplasm but not in the nucleus of control cells. No interactions with caspase-2L were observed for P-αB-crystallin-Ser19, -Ser45 or -Ser59 in either the cytoplasm or the nucleus. Importantly, MGO treatment abolished the interaction between unphosphorylated αB-crystallin and caspase-2L. This dissociation began at 24 h post-treatment and became more evident at 48 h post-treatment, indicating that release of caspase-2L from αB-crystallin precedes the production of caspase-2L cleavage products in the cytoplasm (Fig. 4B). In contrast to caspase-2L, caspase-7 and -3 interacted with αB-crystallin in both the cytoplasm and nuclei of control cells (Fig. 4C, D). Whereas both caspase-7 and -3 interacted with phosphorylated αB-crystallins in the nucleus, they interacted with only unphosphorylated αB-crystallins in the cytoplasm. The interactions between αB-crystallin and caspase-7 or -3 were generally decreased in the cytoplasm and the nuclei of ARPE-19 cells treated with MGO. However, the interactions between phosphorylated αB-crystallin and caspase-7 or -3 in the nucleus were generally sustained or even increased during MGO treatment (Fig. 4C, D). Confocal microscopy revealed that caspase-7 and -3 were diffusely distributed in the nuclei of control cells. Importantly, MGO treatment induced relocalization of caspase-7 and -3 into SC35 speckles in which phosphorylated αB-crystallin was also located (Fig. 4E–G). These data suggest that although αB-crystallin released caspases 2L, -3 and -7 in the cytoplasm following MGO treatment, phosphorylated αB-crystallin sustains its association with caspase-3 and -7 in SC35 speckles. This association may represent a last line of defense in resisting MGO-induced apoptosis.

### αB-crystallin Inhibits the Activation of Caspase-2 and -7 in the Nuclei of Control ARPE-19 Cells

To directly assess the function of each αB-crystallin phosphorylation site, we used site-directed mutagenesis to convert these serines to alanines. We transiently transfected ARPE-19 cells with either wild-type or mutant *CRYAB* plasmids. Replacing serine Ser19, Ser45 or Ser59 with alanine caused the loss of αB-crystallin from the nucleus (Fig. 5A, B). Transfection with these mutants did not cause a reduction in viability (Fig. 5C). Transfection of these mutants produced notable amounts of caspase-2L cleavage without upregulating caspase-2L RNA transcripts (Fig. 5D). Transfection of these mutants produced the cleavage products of caspase-7 without prior processing of caspase-3 or PARP or the upregulation of PIDD-CC (Fig. 5E). The caspase-7 cleavage products were distributed in the cytoplasm and nucleus; however, the cleavage products of caspase-2L were found only in the cytoplasm. These findings indicate that depletion of αB-crystallin in the nucleus triggers the activation of caspase-7, which may cleave caspase-2L in the cytoplasm. However, caspase-3 cleavage products were not observed in either the cytoplasm or in the nucleus, indicating that the dissociation of nuclear caspase-3 from αB-crystallin appears to be insufficient to induce activation of caspase-3 (Fig. 5F). We next depleted both cytoplasmic and nuclear αB-crystallin using siRNA. Our data indicate that silencing of αB-crystallin produced a caspase-2L cleavage product as well as inducing the degradation of procaspase-7 and the formation of its cleavage product. However, silencing of αB-crystallin was not sufficient to induce the degradation of caspase-3 and PARP or their cleavage products (Fig. 5G). Similarly, silencing αB-crystallin did not upregulate PIDD-CC (Fig. 5G). These data suggest that although the depletion of αB-crystallin in the nucleus and the cytoplasm could initiate apoptosis, additional stimuli are required to execute overt apoptosis. To demonstrate that caspase-7 is activated by the depletion of αB-crystallin in the nucleus and subsequently processes cytoplasmic caspase-2L, caspase-7 was silenced using siRNA, and indeed, caspase-7 siRNA prevented the activation of caspase-2L in S19A, S45A and S59A mutant-expressing cells (Fig. 5H).

### Phosphorylation of αB-crystallin at Serines 19, 45 and 59 Plays a Pivotal Role in Preventing MGO-induced ARPE-19 Apoptosis

We next examined whether replacing the Ser19, Ser45 or Ser59 residues with alanine could enhance rates of cell death in response to MGO. Whereas the interactions between αB-crystallin and caspase-3 and -7 were decreased, the interaction between RAIDD and PIDD was increased in mutant-transfected cells (Fig. 6A). Mutant-expressing cells showed significant reductions in viability at 24 h post-MGO treatment (Fig. 6B). In addition to producing the cleavage products of caspase-2L and -7, the mutant-expressing cells exhibited upregulation of PIDD-CC as well as activation of caspase-3 and PARP at 24 h post-MGO treatment (Fig. 6C). Transfection of these mutants resulted in upregulation of caspase-2L at the mRNA level (Fig. 6D). These data suggest that phosphorylation of αB-crystallin on these serines plays a key role in preventing the induction of apoptosis in ARPE-19 cells. To further investigate this idea, we examined the coordinated activation of caspase-2L, -3 and -7. A specific inhibitor of caspase-2, zVDVAD-fmk, efficiently prevented MGO-induced accumulation of subdiploid apoptotic cells and reduced cell viability. zVDVAD-fmk also prevented MGO-induced degradation of PARP and its cleavage products as well as the upregulation of PIDD-CC. Importantly, zVDVAD-fmk prevented MGO-induced processing of caspase-3. zVDVAD-fmk also partially prevented MGO-induced processing of caspase-7 (Fig. S3A). We next knocked down caspase-7 using siRNA. Caspase-7 siRNA efficiently prevented MGO-induced accumulation of subdiploid apoptotic cells and reductions in cell viability. In addition, caspase-7 siRNA abolished MGO-induced processing of caspase-2L. Caspase-7 siRNA also prevented the processing of procaspase-3 and upregulation of PIDD-CC (Fig. S3B). The specific inhibitor of caspase-3, Ac-DMQD-CHO, also efficiently prevented MGO-induced accumulation of subdiploid apoptotic cells and reductions in cell viability. Ac-DMQD-CHO abolished MGO-induced processing of caspase-2L. Ac-DMQD-CHO also prevented the processing of procaspase-7 and PIDD-CC upregulation (Fig. S3C). Taken together, these findings indicate that caspase-2, -3 and -7 coordinately activate each other in MGO-treated ARPE-19 cells, ultimately leading to apoptosis.

## Discussion

We believe the most intriguing finding of our study is that αB-crystallin protects RPE cells from MGO-induced apoptosis by interacting with caspase subtypes in the cytoplasm and nucleus. To date, there have been only a few previous reports showing that αB-crystallin prevents apoptosis through interactions with procaspase-3 and partially processed procaspase-3 [21], [22]. Here, for the first time, we observed that αB-crystallin interacts with several caspase subtypes and that MGO treatment induces their dissociation. Our findings indicate that these interactions play a pivotal role in the cascade of caspases that occurs during apoptosis in ARPE-19 cells. Importantly, we observed that caspase-7 dissociates from αB-crystallin in the nucleus and processes caspase-2. This finding is consistent with the results of a previous study showing that processing of caspase-2 occurs downstream of caspase-7 in certain circumstances [23]. However, even considering the new information presented in this study, the precise hierarchical ordering of caspases in apoptotic ARPE-19 cells remains unclear. Exactly how this process is regulated by αB-crystallin warrants further investigation.

Here, we demonstrate that MGO-induced apoptosis of ARPE-19 cells is mediated by caspase-2. Caspase-2 has been previously proposed as an initiator caspase; however, compared to other caspases, the link to between caspase-2 and apoptosis had not been firmly established [24]–[26]. We also show that MGO modulates caspase-2 splicing in ARPE-19 cells. Expression of caspase-2 is modulated by alternative promoters and splicing [24], [27]. Our data support the notion that MGO upregulates the expression of caspase-2L RNA transcripts. The present study also suggests that MGO upregulates SC35 and ASF/SF2, which is consistent with the suggested roles for splicing factors in apoptotic regulation [28]. Because SC35 speckles are sites of storage and recycling of splicing factors [29], the specific localization of αB-crystallin to nuclear speckles suggests a role for αB-crystallin in splicing or in protection of the splicing machinery [30]. We also observed that depletion of αB-crystallin from the nucleus via transfection of αB-crystallin mutants upregulated SC35 and ASF/SF2 (data not shown). Thus, the regulation of caspase-2 splicing by MGO appears to be accomplished through modulation of αB-crystallin in SC35 speckles. Additionally, we demonstrated that the interaction between αB-crystallin and PIDDosome components plays a role in protecting ARPE-19 cells from apoptosis. Activation of caspase-2 occurs within a complex called the PIDDosome, which contains the death domain-containing protein PIDD and the adaptor protein RAIDD (or potentially other adaptors) [31]. Within this complex, PIDD promotes caspase-2 processing [31]. However, PIDD may not be essential for caspase-2 activation, and an alternative PIDDosome-independent DNA-damage response mechanism of caspase-2 activation is known to exist in mammals [32], [33]. We showed that the release of RAIDD and PIDD from αB-crystallin and the subsequent release of caspase-2L from αB-crystallin induce the formation of PIDDosomes and play a role in the initiation of apoptosis.

This study further demonstrated that phosphorylated αB-crystallin interacts with caspases in the nucleus, preventing the dissociation of caspases from αB-crystallin. Phosphorylation of αB-crystallin is known to occur primarily on serines 19, 45 and 59 [34], [35]. Phosphorylation of Ser59 is crucial for nuclear import, and phosphorylation of Ser45 is required for speckle localization [3]. The present study also demonstrated that phosphorylation of αB-crystallin on these serine residues determines its nuclear localization and anti-apoptotic role in ARPE-19 cells. Previous reports have shown that Hsps and alpha-crystallin are particularly vulnerable to MGO modification [20], [36], [37] and that MGO induces apoptosis by altering the phosphorylation status of various proteins [38], [39]. Here, we observed that MGO treatment altered the phosphorylation status of αB-crystallin and increased its rate of nuclear import; these effects were observed in nuclear SC35 speckles and exerted an anti-apoptotic function [40].

Although we report here the mechanism underlying the αB-crystallin-mediated anti-apoptotic activity in response to MGO *in vitro*, it is not yet certain that the mechanism described here accurately reflect the protective effects of αB-crystallin against MGO toxicity in RPE cells *in vivo.* However, when designing preventive or therapeutic strategies to modulate the loss of RPE cells in AMD or to efficiently induce apoptosis in PVR patients, the mechanism of αB-crystallin-mediated RPE protection against MGO toxicity should be cautiously considered.

## Supporting Information

Figure S1 **Dose- and time-dependent decrease in viability of ARPE-19 cells in response to methylglyoxal (MGO).** Viability was assessed using an automated trypan blue exclusion assay with a cell counter (* *P*<0.05 or ** *P*<0.01). (A) Viability of ARPE-19 cells treated with different doses of MGO for 48 h. (B) Changes in cell viability over time in response to 2 mM MGO.
(TIF)

Figure S2 **Methylglyoxal (MGO)-induced apoptosis of ARPE-19 cells.** ARPE-19 cells were treated with 2 mM MGO. **(**Ctrl, control) (A) DNA electrophoresis and PFGE. Although conventional agarose gel electrophoresis did not reveal ladder-like DNA fragments (left panel), the disintegration of nuclear DNA into fragments ranging in size from 100 kbp to 2 Mbp was revealed by PFGE (right panel). M1, low molecular weight marker; M2, high molecular weight marker. (B) Nuclear morphology revealed by Hoechst staining. Nuclear condensation was observed following treatment with 2 mM MGO for 48 h. (C) Representative histograms indicating cell cycle progression and induction of apoptosis (Apo, the percentage of the population undergoing apoptosis). An accumulation of subdiploid apoptotic cells was observed in cells treated with MGO. (D) A western blot assay of apoptosis-related proteins. MGO induced the degradation of procaspase-3, -7 and PARP, as well as the formation of their respective cleavage products. MGO downregulated the expression of survivin, XIAP and Bcl-2 in a time dependent manner (β-actin was used as a loading control). (E) Flow cytometry results indicating the reduction of mitochondrial membrane potential (MMP) in ARPE-19 cells treated with MGO. (F) Confocal microscopy images showing the subcellular localization of cytochrome c. The release of cytochrome c from mitochondria was observed at 48 h after treatment with 2 mM MGO (PI, propidium iodide; Cyto C, cytochrome c). (G) Protection of ARPE-19 cells from MGO-induced cell death. Pretreatment with 100 µM zVAD-fmk protected cells from MGO-induced apoptosis (zVAD, zVAD-fmk). ** *P*<0.01.
(TIF)

Figure S3 **Caspase-2 -7 and -3 are coordinately activated in ARPE-19 cells treated with MGO.** (A) The effect of the specific caspase-2 inhibitor zVDVAD-fmk. zVDVAD-fmk prevented the MGO-induced reduction in viability and accumulation of subdiploid apoptotic cells. zVDVAD-fmk prevented the activation of caspase-3 and -7 in MGO-treated ARPE-19 cells. zVDVAD-fmk prevented the upregulation of PIDD-CC. (B) The effect of caspase-7 siRNA. Caspase-7 siRNA prevented MGO-induced reduction of viability and accumulation of subdiploid apoptotic cells. Caspase-7 siRNA prevented the activation of caspase-2 and -3 and the upregulation of PIDD-CC in MGO-treated ARPE-19 cells. (C) The effect of the specific caspase-3 inhibitor Ac-DMQD-CHO (Calbiochem, San Diego, CA, USA). Ac-DMQD-CHO prevented the MGO-induced reduction in viability and accumulation of subdiploid apoptotic cells. Ac-DMQD-CHO prevented the activation of caspase-2 and -3 and the upregulation of PIDD-CC in MGO-treated ARPE-19 cells. β-actin was used as a loading control. ** *P*<0.01. See Figure 1 for other definitions.
(TIF)
