# Supplementary figures and images for: Cytoplasmic and Nuclear Anti-Apoptotic Roles of αB-Crystallin in Retinal Pigment Epithelial Cells
Source: PLoS One. 2012 Sep 26;7(9):e45754. doi: 10.1371/journal.pone.0045754 (PMC3458930; doi:10.1371/journal.pone.0045754)

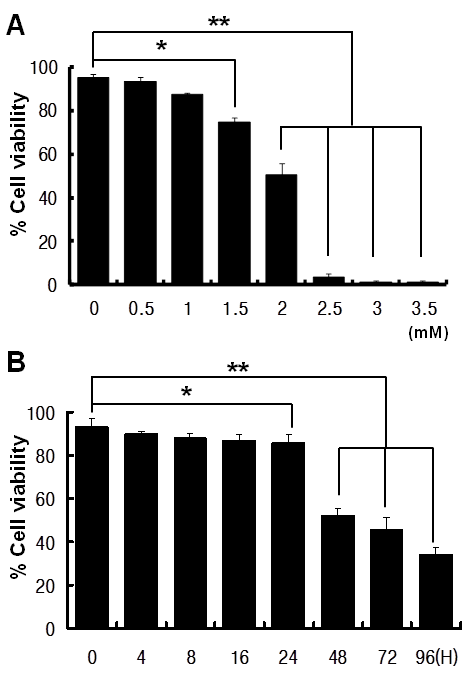

Supplement: Figure S1 — Dose- and time-dependent decrease in viability of ARPE-19 cells in response to methylglyoxal (MGO). Viability was assessed using an automated trypan blue exclusion assay with a cell counter (* P<0.05 or ** P<0.01). (A) Viability of ARPE-19 cells treated with different doses of MGO for 48 h. (B) Changes in cell viability over time in response to 2 mM MGO. (TIF) [file pone.0045754.s001.tif]

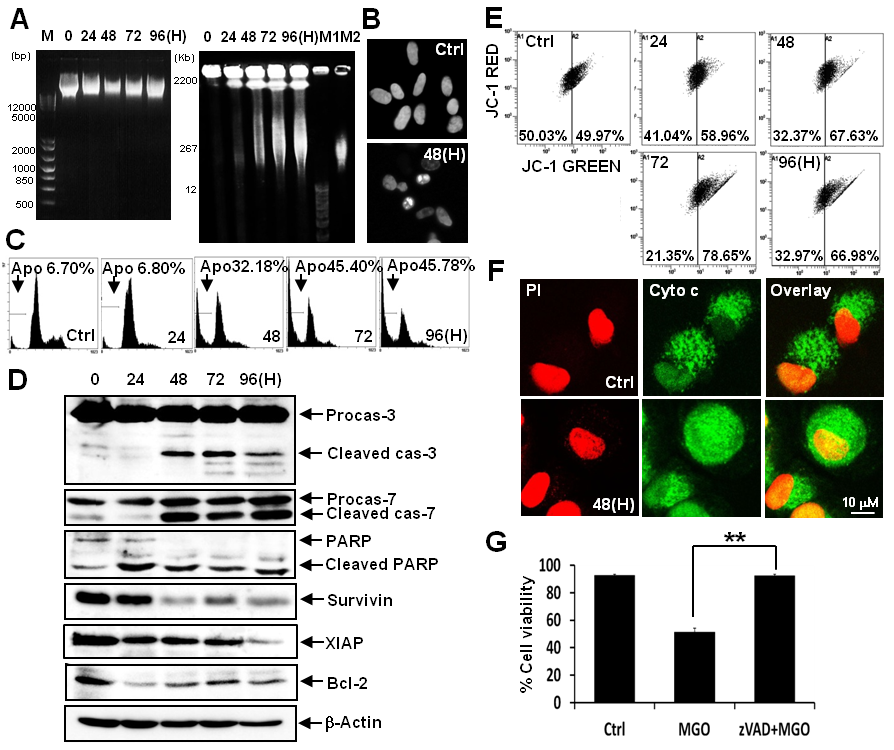

Supplement: Figure S2 — Methylglyoxal (MGO)-induced apoptosis of ARPE-19 cells. ARPE-19 cells were treated with 2 mM MGO. (Ctrl, control) (A) DNA electrophoresis and PFGE. Although conventional agarose gel electrophoresis did not reveal ladder-like DNA fragments (left panel), the disintegration of nuclear DNA into fragments ranging in size from 100 kbp to 2 Mbp was revealed by PFGE (right panel). M1, low molecular weight marker; M2, high molecular weight marker. (B) Nuclear morphology revealed by Hoechst staining. Nuclear condensation was observed following treatment with 2 mM MGO for 48 h. (C) Representative histograms indicating cell cycle progression and induction of apoptosis (Apo, the percentage of the population undergoing apoptosis). An accumulation of subdiploid apoptotic cells was observed in cells treated with MGO. (D) A western blot assay of apoptosis-related proteins. MGO induced the degradation of procaspase-3, -7 and PARP, as well as the formation of their respective cleavage products. MGO downregulated the expression of survivin, XIAP and Bcl-2 in a time dependent manner (β-actin was used as a loading control). (E) Flow cytometry results indicating the reduction of mitochondrial membrane potential (MMP) in ARPE-19 cells treated with MGO. (F) Confocal microscopy images showing the subcellular localization of cytochrome c. The release of cytochrome c from mitochondria was observed at 48 h after treatment with 2 mM MGO (PI, propidium iodide; Cyto C, cytochrome c). (G) Protection of ARPE-19 cells from MGO-induced cell death. Pretreatment with 100 µM zVAD-fmk protected cells from MGO-induced apoptosis (zVAD, zVAD-fmk). ** P<0.01. (TIF) [file pone.0045754.s002.tif]

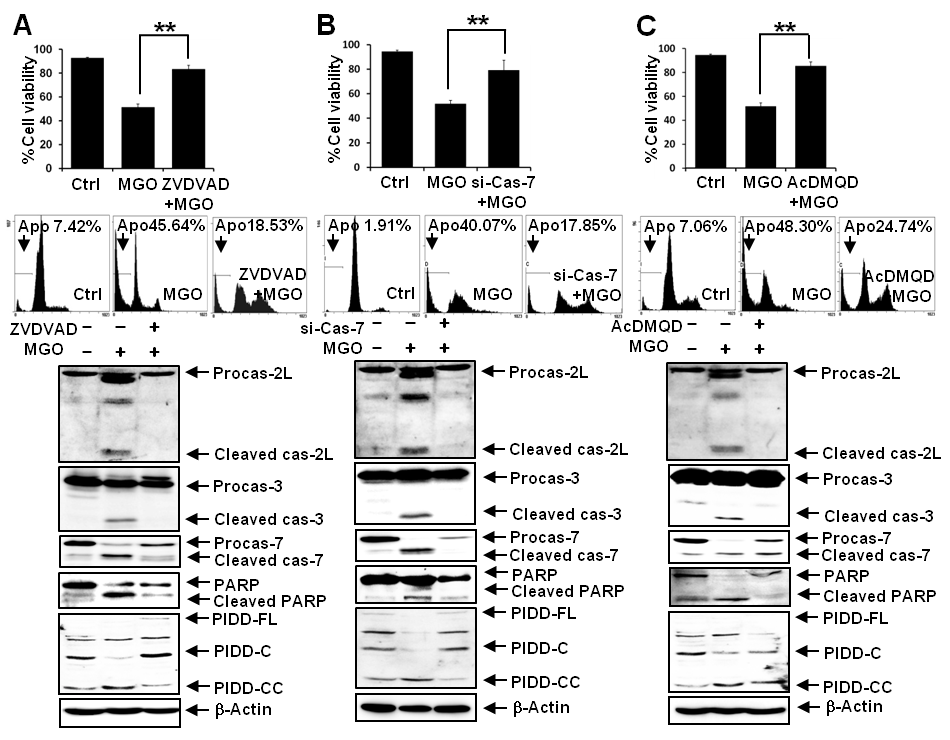

Supplement: Figure S3 — Caspase-2 -7 and -3 are coordinately activated in ARPE-19 cells treated with MGO. (A) The effect of the specific caspase-2 inhibitor zVDVAD-fmk. zVDVAD-fmk prevented the MGO-induced reduction in viability and accumulation of subdiploid apoptotic cells. zVDVAD-fmk prevented the activation of caspase-3 and -7 in MGO-treated ARPE-19 cells. zVDVAD-fmk prevented the upregulation of PIDD-CC. (B) The effect of caspase-7 siRNA. Caspase-7 siRNA prevented MGO-induced reduction of viability and accumulation of subdiploid apoptotic cells. Caspase-7 siRNA prevented the activation of caspase-2 and -3 and the upregulation of PIDD-CC in MGO-treated ARPE-19 cells. (C) The effect of the specific caspase-3 inhibitor Ac-DMQD-CHO (Calbiochem, San Diego, CA, USA). Ac-DMQD-CHO prevented the MGO-induced reduction in viability and accumulation of subdiploid apoptotic cells. Ac-DMQD-CHO prevented the activation of caspase-2 and -3 and the upregulation of PIDD-CC in MGO-treated ARPE-19 cells. β-actin was used as a loading control. ** P<0.01. See Figure 1 for other definitions. (TIF) [file pone.0045754.s003.tif]
